# Prevalence and Multilocus Genotyping of *Giardia duodenalis* in Donkeys in Shanxi Province, North China

**DOI:** 10.3390/ani13243771

**Published:** 2023-12-06

**Authors:** Wen-Wei Gao, Shuo Zhang, Tian-Hong Zhang, Han-Dan Xiao, Nan Su, Meng-Fan Tao, Ze-Xuan Wu, Ze-Dong Zhang, Xing-Quan Zhu, Shi-Chen Xie

**Affiliations:** 1 Laboratory of Parasitic Diseases, College of Veterinary Medicine, Shanxi Agricultural University, Jinzhong 030801, China; shuoshuozhang0828@163.com (S.Z.); a1185927932@163.com (T.-H.Z.); 15166873600@163.com (H.-D.X.); sunan1228@163.com (N.S.); wuzexuan0602@163.com (Z.-X.W.); zzd18203541696@163.com (Z.-D.Z.); xingquanzhu1@hotmail.com (X.-Q.Z.); 2 Shanxi Key Laboratory of Integrated Pest Management in Agriculture, College of Plant Protection, Shanxi Agricultural University, Taiyuan 030031, China; taomf615@163.com; 3 Key Laboratory of Veterinary Public Health of Higher Education of Yunnan Province, College of Veterinary Medicine, Yunnan Agricultural University, Kunming 650201, China

**Keywords:** *Giardia duodenalis*, donkey, prevalence, assemblage, Shanxi Province

## Abstract

**Simple Summary:**

*Giardia duodenalis* is a common zoonotic intestinal protozoan and causes significant economic losses and potential risks to public health. China is among the several major producers of donkeys worldwide, with approximately 2.6 million donkeys being reared in 2019, but studies on *G. duodenalis* infection in donkeys were limited. Thus, in order to better understand the molecular epidemiology of *G. duodenalis* in donkeys in China, 815 fecal samples were collected from donkeys in three representative geographical locations in Shanxi Province, North China, to determine the presence and genotypes of *G. duodenalis* based on three established loci (*tpi*, *bg*, and *gdh*). A total of 137 out of 815 fecal samples were detected as *G. duodenalis*-positive with an overall prevalence of 16.81%. Among the detected assemblages A, B, and E, the most predominant assemblage was B in the three study areas. Three MLGs (MLG-novel-1 to 3) were identified with multilocus genotypes (MLGs) analysis and validated by a phylogenetic tree. This is the first report of *G. duodenalis* infection in donkeys in Shanxi Province, which not only enhances our understanding of the genetic diversity of *G. duodenalis* in donkeys in China but also provides essential baseline data for the prevention and control of *G. duodenalis* infection donkeys in the study areas.

**Abstract:**

*Giardia duodenalis* is a ubiquitous flagellated protozoan, causing significant economic losses to animal husbandry and posing threats to public health. China ranks the world’s sixth largest major producer of donkeys, rearing approximately 2.6 million donkeys in 2019, but limited investigation of *G. duodenalis* prevalence has been conducted in the past, and it is yet to be known whether donkeys in Shanxi Province are infected with *G. duodenalis*. In the present study, a total of 815 fecal samples collected from donkeys in representative regions of Shanxi Province, North China, were examined for *G. duodenalis* using nested PCR. Then, the assemblages and multilocus genotypes (MLGs) were examined based on three established loci: namely, β-giardin (*bg*), triosephosphate isomerase (*tpi*), and glutamate dehydrogenase (*gdh*). The overall prevalence of *G. duodenalis* in donkeys in Shanxi Province was 16.81% (137/815). The region was identified as the main risk factor for the observed difference in *G. duodenalis* prevalence in donkeys among the three study areas (χ^2^ = 21.611, *p* < 0.001). Assemblages A, E, and B were identified, with the latter as the predominant assemblage. Three MLGs (MLG-novel-1 to 3) were formed based on sequence variation among the three loci. The present study reveals the presence of *G. duodenalis* in donkeys in Shanxi Province, North China, for the first time, which not only enriches the data on the distribution of *G. duodenalis* in donkeys in China but also provides useful baseline data for planning control strategies against *G. duodenalis* infection in the sampled areas.

## 1. Introduction

*Giardia* is a genus of intestinal flagellates infecting a wide variety of vertebrates, including horses and donkeys [1,2]. According to the current knowledge, eight species of *Giardia* with different host preferences are retained as valid, including *Giardia cricetidarum*, *Giardia duodenalis*, *Giardia muris*, *Giardia agilis*, *Giardia psittaci*, *Giardia microti*, *Giardia ardeae,* and *Giardia peramelis* [3,4,5]. *G. duodenalis* (syn. *G. intestinalis* and *G. lamblia*) is the only species responsible for all human infections, although it is also detected in many other mammals [6]. It contributes to approximately 280 million cases of human diarrhea every year [7] and infects over 40 different animal species [8]. Asymptomatic or mild infection usually occurs in most people, whereas chronic *Giardia* infection also appears in individuals who suffer from food allergies, irritable bowel syndrome (IBS), chronic fatigue syndrome, and arthritis [9]. Similarly, once infected with *G. duodenalis*, equine animals, including donkeys and horses, exhibited asymptomatic to severe clinical symptoms, such as abdominal pain, anemia, wasting, stunted growth and development, and even fatality [10,11].

*Giardia duodenalis* has been categorized as a minimum of eight assemblages or genotypes (A–H) based on genetic analysis [12]. Among them, assemblages A and B are commonly detected in humans and other mammals, whereas assemblages C, D, E, and F have also been sporadically reported in humans [4]. Meanwhile, assemblages G and H exhibit specificity to particular animal hosts, such as murines and seals [4]. Up to now, three assemblages (A, B, and E) have been detected in donkeys [13,14,15], suggesting that donkeys might be a potential reservoir for the transmission of *G. duodenalis* to humans or other animal hosts. In addition to assemblages A, B, and E, other assemblages, such as G, have been notably reported in horses [15,16]. Donkeys are closely related to horses; a better understanding of the genetic diversity and population genetic structure of *G. duodenalis* in animals of the genus *Equus* could contribute to the execution of precise control strategies against *G. duodenalis* infection in equine animals. 

Domesticated from wild asses, donkeys are reared worldwide as draft animals as well as for food, medicine, and cosmetics purposes [17]. In fact, donkey meat is considered to be a superior nutritional source for human consumption due to its abundant essential and nonessential amino acids and lower fat content [18]. Donkey skins can be used to produce the traditional Chinese medicine “donkey-hide gelatin”, which is widely accepted by Chinese people [19]. In addition, a previous study indicated that donkey milk has multifunctions, such as boosting immunity and aiding digestion [20]. For example, donkey milk was used in northwestern China to treat lung and stomach diseases [21]. 

China is ranked the sixth largest major producer of donkeys, with approximately 2.6 million donkeys in 2019 worldwide [17], but limited surveys of *G. duodenalis* prevalence have been carried out only in a few Chinese provinces in the past [13,14], and no information on *G. duodenalis* prevalence in donkeys in Shanxi Province is available. Shanxi Province is located in the west of the North China Plain and has a temperate continental monsoon climate [22]. Due to its typical topography of loess-covered mountain plateau, Shanxi Province has a significant climate and precipitation difference and is greatly affected by the terrain, with an annual average temperature of from 4.2 to 14.2 °C and annual precipitation of from 358 to 621 mm from north to south [22,23]. A better understanding of *G. duodenalis* genetic diversity in donkeys in Shanxi Province under the unique topography, climate, and precipitation conditions has important implications for the better prevention and control of *G. duodenalis* in donkeys in Shanxi Province. Therefore, the objectives of the present investigation were to reveal the prevalence and genotypes of *G. duodenalis* in donkeys in Shanxi Province using a PCR-based molecular approach combined with multilocus genotyping based on the loci of glutamate dehydrogenase (*gdh*), triosephosphate isomerase (*tpi*), and β-giardin (*bg*) genes.

## 2. Materials and Methods

### 2.1. Sample Collection

From April to May 2023, a total of 815 fresh fecal samples were collected from donkeys in three cities as follows: Jinzhong (*n* = 81), Linfen (*n* = 363), and Datong (*n* = 371). The uppermost portion of freshly excreted donkey feces was carefully sampled to minimize the potential contamination of soil and water by *G. duodenalis*. According to the reported *G. duodenalis* prevalence in donkeys in China, at least 97 to 229 fecal samples need to be collected following the formula given in a previous report [24], and approximately 30–50% of donkeys on each donkey farm in the same region were collected to ensure that the samples were representative. All samples were divided into two age groups (older donkeys ≥ 3 years old and younger donkeys < 3 years old) and two sex groups (male and female). Each fecal sample was individually collected into a sterile glove and labeled with relevant details, including age, sex, and geographic location. These samples were then transferred into a container with ice packs for transportation to the Laboratory of Parasitic Diseases at the College of Veterinary Medicine, Shanxi Agricultural University. Afterwards, the samples were immediately stored in a freezer at −20 °C for preservation until used for genomic DNA extraction.

### 2.2. DNA Extraction and PCR Amplification

Genomic DNA was extracted from 200 mg of each fecal sample using the commercial E.Z.N.A.^®^ Stool DNA Kit (Omega, Biotek Inc., Norcross, GA, USA) following the manufacturer’s specifications. The overall prevalence of *G. duodenalis* in donkeys was determined using nested PCR targeting the *bg*, *tpi*, and *gdh* gene individually, and the overall prevalence was calculated according to a previous report [25]. Briefly, each PCR mixture consisted of 2 μL of dNTPs, 2.5 μL of 10 × PCR Buffer (Mg^2+^ free), 25 mM of MgCl_2_, 1.25 U of *Ex*-Taq (Takara, Dalian, China), 1 μL of each primer, 2 μL of DNA template, and 14.75 μL of ddH_2_O to achieve a total volume of 25 μL. The PCR reaction condition and parameters for the amplification of the three loci were based on previously reported methods and are summarized in Table 1 [2,26,27]. Both negative (reagent-grade water) and positive controls (verified DNA of *G. duodenalis* with sequencing) were added to each PCR assay to ensure the reliability of the results. All secondary PCR products were electrophoresed on ethidium bromide-stained 1.5% agarose gels and visualized under UV light.

### 2.3. Sequencing and Phylogenetic Analysis

To validate the accuracy of the positive secondary PCR amplicons, they were bisequenced by Sangon Biotech Co., Ltd. (Shanghai, China). The obtained sequences were then aligned with known reference sequences in the GenBank database for the identification of genotypes. The representative sequences obtained in the present study were submitted to the GenBank database with the following accession numbers: OR636105 to OR636116 for the *bg* gene, OR497193 to OR497199 for the *gdh* gene, and OR497200 to OR497210 for the *tpi* gene. A phylogenetic analysis of *G. duodenalis* based on multilocus genotypes (MLGs) was constructed using the Neighbor-joining (NJ) method in MEGA 7.0 software. The Kimura 2-parameter model and 1000 bootstrap replicates were employed to calculate the genetic distances and evaluate the reliability of clusters, respectively.

### 2.4. Statistical Analysis

The chi-square (χ2) test in SPSS 26.0 (SPSS Inc., Chicago, IL, USA) was used to analyze the relationships between *G. duodenalis* prevalence and risk factors, including geographical location, age, and sex. Odds ratios (ORs) and 95% confidence intervals (95% CIs) were calculated to investigate the magnitude of the association between prevalence and the risk factors considered above. A statistically significant difference was approved when the *p*-value was less than 0.05.

## 3. Results

### 3.1. Prevalence of G. duodenalis in Donkeys

Based on the *bg*, *gdh*, and *tpi* gene loci, a total of 126, 36, and 74 fecal samples were amplified and detected as *G. duodenalis*, respectively, giving a total prevalence of 16.81% (137/815) (Table 2). The *G. duodenalis* infection existed in each examined city, with a significant difference in *G. duodenalis* prevalence among three surveyed cities (*p* < 0.001). Specifically, the prevalence of *G. duodenalis* from highest to lowest was in Linfen City (23.42%), Datong City (12.67%), and Jinzhong City (7.41%). For the different age groups, the prevalence of *G. duodenalis* in donkeys ≥ 3 years (17.97%) was higher than that in donkeys less than 3 years (13.55%); however, no statistically significant difference was detected (*p* = 0.139). When grouped by sex, no significant difference in *G. duodenalis* prevalence was observed between males (16.67%) and females (16.83%).

### 3.2. Identification and Distribution of G. duodenalis Assemblages

Among 137 *G. duodenalis*-positive fecal samples, sequence analysis identified three assemblages (A, B, and E) at three gene loci within both age group and sex group. Notably, all three assemblages (A, B, and E) were detected in Linfen City, whereas only one and two assemblages were detected in Jinzhong City and Datong City, respectively (Table 3). Assemblage B was the most prevalent genotype, being found in 70 fecal samples in every region, age, and sex group. Meanwhile, ten samples were positive for assemblage A and 18 samples for assemblage E.

### 3.3. Analysis of G. duodenalis Subtypes and MLGs

At the *bg* locus, one known subtype and one novel subtype (designated as A-novel-1) were detected within the assemblage of A-positive samples (Table S1). The known sequence assemblage was identical to the GenBank reference sequence with accession number KJ027408. Meanwhile, six subtypes of assemblage B were identified, including one known assemblage B subtype (B3) and five novel assemblage B subtypes (designated as B-novel-1 to B-novel-5) (Table S1). In addition, four novel subtypes of assemblage E were first identified, all of which have not been reported previously (Table S1).

At the *gdh* locus, one known subtype of assemblage A (A1) was identified (Table S2). Meanwhile, three types of sequences were present within the assemblage of B-positive samples, which have not been reported previously (Table S2). In addition, three novel subtypes of assemblage E were identified and designated as E-novel-1 to E-novel-3 (Table S2).

**Figure 1:**
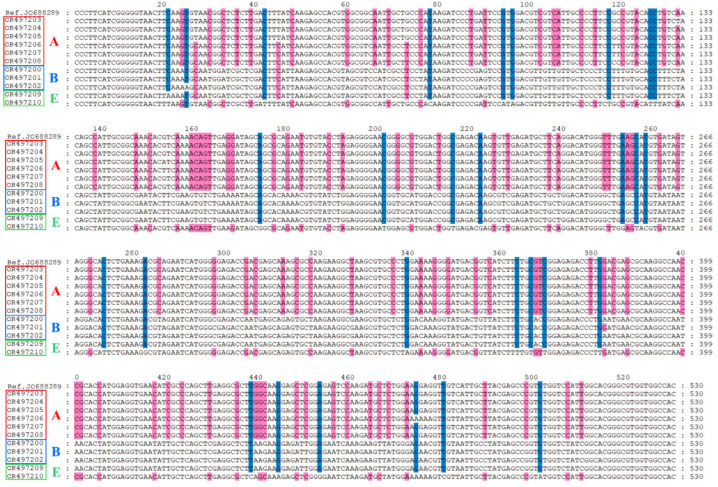
Multiple sequence alignment in *tpi* gene within *Giardia duodenalis* assemblages A, B, and E in donkeys. GenBank reference sequence with accession number: JQ688289.

At the *tpi* locus, six subtypes of assemblage A were identified, including one known subtype of assemblage A (A1) and five novel subtypes of assemblage A (A-novel-1 to A-novel-5) (Figure 1). Meanwhile, four subtypes of assemblage B were identified; one was B4, and the others were named B-novel-1 to B-novel-3, respectively (Figure 1). Additionally, one novel subtype of assemblage E (E-novel-1) was identified (Figure 1).

**Figure 2:**
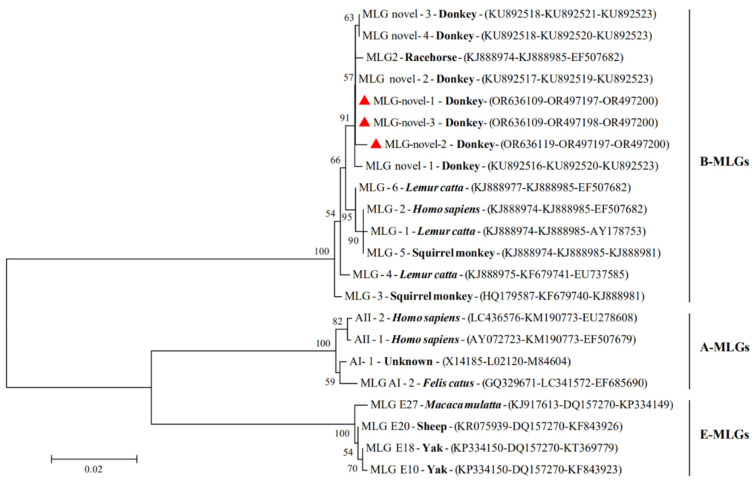
Phylogenetic tree of *Giardia duodenalis* multilocus genotypes (MLGs). The bootstrapping used the Kimura 2-parameter model and 1000 bootstrap replicates. Isolates identified in this study are indicated by red triangles.

Multilocus genotyping formed three different assemblage B MLGs based on three gene loci (Table 4). Among 137 *G. duodenalis*-positive samples, 22 samples were simultaneously amplified at all three intra-assemblage variation genes (*bg*, *gdh*, and *tpi*), including 5 samples identified as assemblage B at three loci, and were designated as MLG-novel-1 (*n* = 3), MLG-novel-2 (*n* = 1), and MLG-novel-3 (*n* = 1). Additionally, 23 samples were identified as mixed assemblages, including the mixed infection of assemblage B and E (*n* = 11), followed by the mixed infection of assemblage A and B (*n* = 10) and the mixed infection of assemblage A and E (*n* = 2); there was no significant regional bias among three cities (Table S3). Phylogenetic relationships of assemblage B MLGs of the obtained sequences in this study and the reported sequences are shown in Figure 2. The three MLGs from donkeys detected in this study formed into one cluster, which was closely related to those previously reported in donkeys and horses [13].

## 4. Discussion

As a significant intestinal parasite prevalent in a variety of animals, including sheep, goats, dogs, and cats [28,29,30], *G. duodenalis* prevalence has not been extensively investigated in donkeys. In the present study, molecular examination of *G. duodenalis* in 815 fecal samples collected from donkeys in Shanxi Province, North China revealed an overall *G. duodenalis* prevalence of 16.81%, which was higher than that in donkeys in Jilin (10.42%), Liaoning (13.79%) [13], Inner Mongolia (6.82%), and Xinjiang (14.76%) [14] but lower than that in Shandong province (18.27%) [13]. Moreover, our results showed that *G. duodenalis* prevalence in donkeys in Linfen City (23.42%) was higher than that in Datong City (12.67%) and Jinzhong City (7.41%). The prevalence of *G. duodenalis* in donkeys exhibited significant regional variation in the present study, consistent with a previous survey that was conducted in northern China [14]. Previous research has demonstrated that diverse temperatures and humidity levels could be conducive to the survival and dissemination of *G. duodenalis* cysts [31,32]. Linfen City is situated in the southern part of Shanxi Province and is characterized by elevated average temperatures and a more humid climate, which may contribute to the higher *G. duodenalis* prevalence in donkeys than the other two cities. The previous reports indicated that higher *G. duodenalis* prevalence was generally detected in younger donkeys due to their unhealthy immune system, but this result is not consistent with the two previous reports in China [13,14]. Compared to male donkeys, a higher *G. duodenalis* prevalence was observed in the female donkeys, contradicting the results of a previous investigation on horses in Turkey [33]. In two other studies, no significant difference in *G. duodenalis* prevalence was detected between the different age or sex factors [34,35]. We speculated that limited sample size and sampling sites may have contributed to these prevalence variations; thus, large-scale surveys need to be conducted in the future to further explore the potential linkage between *G. duodenalis* prevalence and risk factors.

Of the eight assemblages of *G. duodenalis*, assemblages A and B exhibit a wide host range [36], whereas the remaining assemblages show a stronger host specificity [28]. Studies have demonstrated that assemblages A and B were two crucial genotypes for human giardiasis, which were also detected in most animals, highlighting the potential zoonotic risk [4]. In addition to assemblages A and B, assemblage E was also detected in this study. Significantly, assemblage B emerged as the dominant assemblage, consistent with the findings of two previous studies reported on horses and donkeys in China [13,14]. However, assemblage G, previously identified in horses [14], was not found in the present study. Further research is warranted to fully understand the prevalence of *G. duodenalis* assemblages in donkeys in China.

Four assemblage A subtypes (AI, AII, AIII, and AIV) have been reported [37]. Comprehensive epidemiological data indicate that human isolates belonged to AI and AII, while animal isolates were mostly AI, AIII, and AIV [38]. Among the four assemblage B subtypes (BI, BII, BIII, and BIV), human isolates are most commonly BIII and BIV, and animal isolates are BI and BII [37]. The present study identified three subtypes: AI, BIII, and BIV, further underscoring the potential for cross-species transmission of *G. duodenalis*. Although assemblage E is typically found in hoofed animals [29], it has also been reported in humans in Australia, Brazil, and Egypt in recent years [39,40,41]. The presence of assemblage E and three subtypes (AI, BIII, and BIV) indicated that *G. duodenalis* infection in donkeys may pose a potential public health risk.

MLG analysis, based on *bg*, *gdh*, and *tpi* gene sequences, is a valuable technique to identify genetic variations within *G. duodenalis* [42,43]. Using this approach, four distinct MLGs in the assemblage B have been reported in donkeys [13]. In the present study, three assemblage B-MLG genotypes were identified, all of which were new assemblage B MLG genotypes. This indicated that assemblage B had a high genetic diversity. Nevertheless, multilocus genotyping data for *G. duodenalis* infecting donkeys is still limited; thus, more molecular epidemiological investigations in donkeys nationwide are needed to deepen our understanding of genetic diversity in *G. duodenalis* from donkeys.

## 5. Conclusions

The present study reveals an overall *G. duodenalis* prevalence of 16.81% in donkeys in Shanxi Province, North China, for the first time. Assemblage B was the predominant assemblage in the examined donkeys among the identified assemblages A, B, and E. The MLGs tool identified three MLGs within assemblage B at *bg*, *tpi*, and *gdh* loci. These findings enhance our understanding of the genetic diversity of *G. duodenalis* in donkeys in China and provide essential baseline data for the prevention and control of *G. duodenalis* infection in donkeys in the study areas.

## Figures and Tables

**Table 1:** Primers and annealing temperatures for PCR amplification of *bg*, *tpi*, and *gdh* genes.

Gene	Primer Name and Sequences	Amplification Procedures	Size
*bg*	BGF1: 5′-AAGCCCGACGACCTCACCCGCAGTGC-3′	94 °C: 5 min; 35 cycles: (94 °C: 30 s, 56 °C: 30 s, 72 °C:1 min); 72 °C: 10 min	
	BGR1: 5′-GAGGCCGCCCTGGATCTTCGAGACGAC-3′		
	BGF2: 5′-GAACGAACGAGATCGAGGTCCG-3′	94 °C: 5 min; 35 cycles: (94 °C: 45 s, 58 °C: 30 s, 72 °C:45 s); 72 °C: 10 min	515 bp
	BGR2: 5′-CTCGACGAGCTTCGTGTT-3′		
*tpi*	ALF1: 5′-TTCCGTRTYCAGTACAACTC-3′	94 °C: 5 min; 35 cycles: (94 °C: 45 s, 55 °C: 45 s, 72 °C:1 min); 72 °C: 10 min	
	ALR1: 5′-ACCTCGTTCTGRGTGGCGCA-3′		
	ALF2: 5′-ATGACYGAGCTYCAGAGGCACGT-3′	94 °C: 5 min; 35 cycles: (94 °C: 45 s, 58 °C: 45 s, 72 °C:1 min); 72 °C: 10 min	530 bp
	ALR2: 5′-GTGGCGCARGGCATGATGCA-3′		
*gdh*	GDH1: 5′-AAATIATGCCTGCTCGTCG-3′	94 °C: 5 min; 35 cycles: (94 °C: 30 s, 52 °C: 45 s, 72 °C:1 min); 72 °C: 10 min	
	GDH2: 5′-CAAACCTTITCCGCAAACC-3′		
	GDH3: 5′-CCCTTCATCGGIGGTAACTT-3′	94 °C: 5 min; 35 cycles: (94 °C: 30 s, 60 °C: 45 s, 72 °C:1 min); 72 °C: 10 min	530 bp
	GDH4: 5′-GTGGCCACCACICCCGTGCC-3′		

**Table 2:** Risk factors associated with the prevalence of *Giardia duodenalis* in donkeys in Shanxi Province, North China.

Risk Factor	Category	No. of Positive/ Tested	Prevalence % (95% CI)	OR (95% CI)	*p*-Value
Age	<3 years	29/214	13.55 (8.97–18.14)	Ref.	0.139
	≥3 years	108/601	17.97 (14.90–21.04)	1.40 (0.90–2.18)	
Region	Jinzhong	6/81	7.41 (1.70–13.11)	Ref.	<0.001
	Linfen	85/363	23.42 (19.06–27.77)	3.82 (1.61–9.09)	
	Datong	46/371	12.67 (9.25–16.09)	1.77 (0.73–4.30)	
Sex	Male	20/120	16.67 (9.99–23.33)	Ref.	0.692
	Female	117/695	16.83 (14.05–19.62)	1.01 (0.60–1.70)	
Total		137/815	16.81 (14.24–19.38)		

**Table 3:** Occurrence of *Giardia duodenalis* genotypes (assemblages A, B, and E) by age, sex, and geographic location.

Factor	Category	Case Count	No. of Positive	Genotypes (*n*)			
				A	B	E	Mixed
Age	<3 years	214	29	1	16	1	11
	≥3 years	601	108	9	54	17	28
Region	Jinzhong	81	6	0	5	0	1
	Linfen	363	85	1	45	18	21
	Datong	371	46	9	20	0	17
Sex	Male	120	20	1	9	1	11
	Female	695	117	9	61	17	28
Total		815	137	10	70	18	39

**Table 4:** Multilocus sequence genotyping of *Giardia duodenalis* based on the *tpi*, *gdh*, and *bg* genes.

Sample ID	Genotypes			MLGs
	bg	gdh	tpi	
JA-8, JB-49, RC-107	B3	B-novel-1	B4	MLG-novel-1
JB-32	B-novel-5	B-novel-1	B4	MLG-novel-2
JA-79	B3	B-novel-2	B4	MLG-novel-3
RC-53, RC-61	A1	A1	B4	Mixed
RC-79	AI	AI	B-novel-1	Mixed
WR-C10	AI	B-novel-1	B4	Mixed
WR-27	B3	A1	B4	Mixed
RC-95, WR-C21	B3	B-novel-1	AI	Mixed
RC-76, JA-22, RC-101, RC-111	B3	E-novel-1	AI	Mixed
JA-25	B3	E-novel-3	B4	Mixed
JA-52	E-novel-1	B-novel-3	B4	Mixed
JA-78, JB-188, B-202, JB-125	E-novel-1	E-novel-2	B4	Mixed

## Data Availability

The data sets supporting the results of this article have been submitted to the GenBank and accession number shown in the article.

## Supplementary Materials

The following supporting information can be downloaded: https://www.mdpi.com/article/10.3390/ani13243771/s1, Table S1: Intra-subtype substitutions in bg gene within assemblages A, B, and E in donkeys.; Table S2: Intra-subtype substitutions in gdh gene within assemblages A, B, and E in donkeys; Table S3. Mixed assemblages of Giardia duodenalis based on tpi, gdh, and bg loci.
