# Supplementary material for: Prevalence and Multilocus Genotyping of Giardia duodenalis in Donkeys in Shanxi Province, North China
Source: Animals (Basel). 2023 Dec 6;13(24):3771. doi: 10.3390/ani13243771 (PMC10740759; doi:10.3390/ani13243771)
Supplement: Supplementary file 1 [file animals-13-03771-s001.zip › Table S1.pdf]

Table S1. Intra-subtype substitutions in *bg* gene within *Giardia duodenalis* assemblages A, B and E in donkeys.

Subtype (*n*)	GenBank	Nucleotide position and substitutions													
A	Ref.	68	317												
	KJ027408	C	C												
A-novel-1 (12)	OR636107	T	T												
AI (6)	OR636108	C	C												
B	Ref.	166	198	344	443	464	485								
	MG736242	A	G	A	G	G	A								
B3 (69)	OR636109	A	G	A	G	G	A								
B-novel-1 (2)	OR636111	A	G	A	G	G	G								
B-novel-2 (1)	OR636112	A	G	G	G	G	A								
B-novel-3 (1)	OR636113	A	G	A	A	G	A								
B-novel-4 (1)	OR636114	A	G	A	G	A	A								
B-novel-5 (1)	OR636119	T	A	A	G	G	G								
E	Ref.	71	83	140	158	173	182	200	239	251	257	290	401	416	443
	MK610389	A	G	G	T	A	A	T	A	T	A	T	G	C	G
E-novel-1 (1)	OR636105	A	G	G	T	A	A	T	A	T	A	T	G	T	A
E-novel-2 (30)	OR636106	A	G	G	T	A	A	T	A	T	A	T	G	T	G
E-novel-3 (1)	OR636110	G	G	A	C	G	G	C	G	T	G	C	A	C	G
E-novel-4 (1)	OR636116	G	A	A	C	G	A	C	A	C	G	C	A	C	G
