# Supplementary material for: Prevalence and Multilocus Genotyping of Giardia duodenalis in Donkeys in Shanxi Province, North China
Source: Animals (Basel). 2023 Dec 6;13(24):3771. doi: 10.3390/ani13243771 (PMC10740759; doi:10.3390/ani13243771)
Supplement: Supplementary file 1 [file animals-13-03771-s001.zip › Table S2.pdf]

Table S2. Intra-subtype substitutions in *gdh* gene within *Giardia duodenalis* assemblages A, B and E in donkeys.

Subtype (*n*)	Accession number	Nucleotide position and substitutions				
A	Ref.	121	241			
	MK645798	A	A			
AI (9)	OR497193	A	A			
B	Ref.	101	480	481		
	OQ94788	G	A	C		
B-novel-1 (12)	OR497197	A	A	C		
B-novel-2 (1)	OR497198	A	A	G		
B-novel-3 (2)	OR497199	A	G	C		
E	Ref.	24	84	371	472	489
	OP271725	T	C	T	G	G
E-novel-1 (4)	OR497194	C	C	T	G	A
E-novel-2 (7)	OR497195	T	C	T	G	A
E-novel-3 (1)	OR497196	T	T	C	A	A
