# Supplementary material for: Prevalence and Multilocus Genotyping of Giardia duodenalis in Donkeys in Shanxi Province, North China
Source: Animals (Basel). 2023 Dec 6;13(24):3771. doi: 10.3390/ani13243771 (PMC10740759; doi:10.3390/ani13243771)
Supplement: Supplementary file 1 [file animals-13-03771-s001.zip › Table S3-updated.pdf]

Table S3. Mixed assemblages of *Giardia duodenalis* based on the *tpi*, *gdh* and *bg* genes.

**Mixed assemblages (*n*)**	**Genotypes**			**Numbers (*n*)**
	bg	***gdh***	***tpi***	
A+B (10)	A-novel-1	-	B4	4
	B3	AI	-	1
	B3	-	AI	5
A+E (2)	A-novel-1	E-novel-1	-	1
	AI	E-novel-2	-	1
B+E (11)	B3	E-novel-1	-	1
	E-novel-2	B-novel-1	-	2
	E-novel-2	-	B4	8
